# Legislating infrastructure: a state-level model to accelerate endometriosis research and women's health equity

**DOI:** 10.3389/frph.2026.1918764

**Published:** 2026-08-13

**Authors:** E. T. Courtois, L. Roy, J. Kuljancic, K. Mullaj, D. Lockshire, D. E. Luciano

**Affiliations:** 1 The Jackson Laboratory, Bar Harbor, ME, United States; 2 Department of Obstetrics and Gynecology, UConn Health, Farmington, CT, United States

**Keywords:** biorepository, education, endometriosis, legislation—EEC, research

## Abstract

Impact statement: A legislatively mandated, statewide endometriosis biorepository integrated within a multidisciplinary endometriosis focused program demonstrates how policy-driven infrastructure can integrate education, clinical care, harmonized biobanking, and translational research to address persistent gaps in women's health in the long term. The Connecticut Endometriosis Working Group (EWG), a group of legislators, researchers, patients, health care providers, and advocates, was established in 2021. The EWG met monthly to discuss strategies to address endometriosis and propose endometriosis specific legislation which has been a turning point. Public Acts 22–33 and 23–67 directed the establishment, governance, and annual reporting of a coordinated endometriosis data and biorepository program, making Connecticut the first state in the US to pass endometriosis focused legislation, unanimously. The resulting program—EndoRISE, (Endometriosis Research, Innovation, Support and Education) is structured as a public, multi-institutional infrastructure platform linking clinical phenotyping, biospecimen banking, molecular profiling, education, public awareness, and policy engagement. On May 14, 2026, HB5514 was passed making the Endometriosis Working Group a legislatively established working group under the administration of the Committee on Women, Children, Seniors, Equity, and Opportunity. This initiative provides an opportunity to examine whether state-level policy mechanisms can meaningfully influence research capacity in women’s health and particularly address disregarded and underappreciated chronic and systemic female conditions such as endometriosis.

## Introduction

### Infrastructure embedded in statute/law

Endometriosis affects an estimated ∼200 million individuals worldwide and approximately one in 10 female-born individuals. Diagnosis remains delayed by an average of ∼10 years, non-invasive diagnostics are lacking, recurrence after surgery is common, and disease-modifying therapies remain limited and often ineffective (1). Despite its prevalence and socioeconomic burden, public and private research investment in female-dominant (non-cancer related) diseases has historically been misaligned with disease burden (2, 3). The EndoRISE data and biorepository was designed not as a single-site repository but as a legislatively mandated, statewide program co-led by UConn Health and The Jackson Laboratory. The statutory framework required longitudinal phenotyping, standardized operating procedures for biospecimen handling, inclusion of underrepresented populations, facilitation of collaborative research, and public reporting.

The 2022 planning report of EndoRISE articulated a seven-point framework aligned with global harmonization standards. Subsequent EndoRISE annual reports document implementation, including development of dual-coded privacy safeguards, implementation of dedicated REDCap (6) -based data and sample management systems (ORCA Specimen Tracking, REDCap External Module) (7), standardized tissue processing pipelines, and establishment of a formal sample access review committee. After two years, approximately 7,000 biospecimens had been collected under harmonized protocols (8–11).

The biorepository has now transitioned from infrastructure development to active dissemination. Sample distribution has begun and is reaching both academic laboratories and start-up biotechnology companies, national and international, each with a clear goal to advance endometriosis research, diagnosis, and care. This shift from collection to external utilization represents a key operational milestone and enables independent validation, hypothesis testing, and diagnostic development beyond the originating institutions. Embedding infrastructure within statute provides continuity and accountability, although long-term impact will depend on sustained funding and measurable research outputs (4, 5).

## Harmonization and analytical readiness

A defining feature of the EndoRISE biorepository is its alignment with the World Endometriosis Research Foundation Endometriosis Phenome and Biobanking Harmonization Project (WERF-EPHect) (8–11). Standardized phenotyping, surgical annotation, and sample processing are essential in a disease characterized by lesion heterogeneity and clinical variability.

In addition, the EndoRISE biorepository was designed with compatibility for emerging molecular technologies, including single-cell and spatial transcriptomics, with the ultimate goal to demonstrate that each biobanked specimen is compatible with the latest advanced molecular biology technology, a key aspect to advance research in this field (12). Such anticipatory planning addresses limitations observed in legacy collections, where earlier protocols restrict future analytic applications. A recent study using EndoRISE repository-derived specimens illustrate the feasibility of high-resolution neuro-immune–stromal mapping in endometriosis lesions (12). These data demonstrate the analytic capacity made possible by standardized, technology-forward biobanking combined with external sample dissemination.

## Clinical–molecular integration

Endometriosis is increasingly recognized as a systemic inflammatory condition with multisystem manifestations (13, 14). Historically, surgical phenotyping and molecular interrogation have occurred in parallel but in disconnected settings. The partnership between UConn Health and the Jackson Laboratory integrates surgical expertise with genomic technologies within a shared governance framework: the Complex Benign Gynecology (CBG) team, led by the clinical PI (Luciano), works symbiotically with the biobanking/molecular team, led by research PI (Courtois). This integration allows clinical annotation, molecular profiling, and data harmonization to occur within a coordinated structure. The initiation of external sample distribution extends this integration to broader research ecosystems, enabling academic groups to investigate pathophysiology, and biotechnology partners to evaluate biomarker platforms, using rigorously phenotyped material (both biospecimen and comprehensive metadata).

## Equity as a structural design principle

Although endometriosis equally affects patients of all ethnic backgrounds disparities in endometriosis diagnosis, referral, and treatment are well documented, with delayed recognition and limited access to specialist care disproportionately affecting marginalized populations (15–17). The governing legislation explicitly mandates representation of historically underrepresented groups, including Black and Latino individuals, gender-diverse persons, and persons with disabilities.

Integral to improving access to specialized care for all individuals affected by endometriosis, EndoRISE's structured education and awareness programs is designed to address diagnostic delay as a systems-level issue with outreach to patients and first line health care providers. The EndoRISE initiative includes continuing education programs for school nurses, advanced nurse practitioners, pediatricians, primary care providers, and general obstetrician gynecologists to educate these providers about endometriosis. In addition, EndoRISE established a consolidated network of advocates, resources, and information on the CTEndoRISE.org website providing the tools to improve access to care.

Embedding inclusion as a statutory requirement, rather than positioning it as a discretionary objective, fundamentally shapes EndoRISE's program design. To operationalize this mandate, participant enrollment was structured across multiple hospital networks throughout Connecticut (CT), spanning urban and suburban settings and encompassing Medicaid, Medicare, and privately insured populations. Eligible patients with suspected endometriosis undergoing laparoscopic surgery as well as control patients undergoing tubal ligations are approached for study enrollment at their pre-operative visit. Due to funding and infrastructure constraints, the biorepository targets enrollment of up to 200 new participants per year: a minimum of 100 from UConn Health, the primary enrolling site located in a suburban area, and up to 50 per year from each of the two remaining participating sites—e.g., St Francis Hospital is located in the urban environment of Hartford. We excluded prisoners, pregnant patients, and patients with HIV, AIDS or other immunodeficiency disorders from the study. Clinical research coordinators inform eligible patients about the possibility of participating in the EndoRISE biorepository at their pre-op visit and gauge interest in learning more. Interested patients then receive full details from the study clinician, including data-sharing information and complete informed consent. To date, three institutions participate in the EndoRISE biorepository, with a total of six providers, including two institutions that were recruited after presenting EndoRISE to their endometriosis surgeons. All follow the same protocol and procedures for participant enrollment and sample collection used at Uconn Health Center for consistency across sites. Enrollment materials and study instruments were translated into additional languages to broaden accessibility and reduce linguistic barriers to participation. In 2025, eligibility expanded to include adolescents and postmenopausal individuals, with additional clinical partnerships under development. In the future we hope to include non-surgical participants and implement at-home collection kits to mitigate selection bias inherent to surgery-based cohorts and improve access for individuals less likely to receive tertiary referral.

With respect to the demographic composition of our enrolled patient population, most participants identified as White (76.8%) while Black participants accounted are for 8.0%, and other (Hispanic and Asian) accounted for 15.2% of the study cohort across all sites. Demographic data for CT (2024) shows a population which is 61% White, 13% Black and 26% Other (Hispanic and Asian). Although our numbers have not yet reflected the demographics of CT, with expansion of tissue collection to other hospitals the intention is to reach all communities. Local awareness and educational events (e.g., annual EndoRISE forum, endometriosis documentary screening, and participating in local public events such as fairs, running events,…) as well as social media presence and media outreach (e.g., TV, podcast, newspaper) spots have helped us to spread the word about EndoRISE and build trust within the Connecticut and endometriosis community. As biospecimens are disseminated to academic and industry investigators, demographic coverage will be essential to ensure that biomarker discovery and therapeutic development are generalizable across populations. Infrastructure that structurally integrates inclusive enrollment and incorporates public outreach and education may reduce inequities in representation; however, ongoing longitudinal evaluation will be necessary to assess whether these programmatic attributes translate into measurable reductions in disparities in diagnosis and care.

## Policy as translational catalyst

State-level initiatives may influence research ecosystems in several ways. First, curated, and harmonized biospecimen collections reduce barriers to entry into the endometriosis research landscape for both academic and pharma/industry investigators. The initiation of distribution to start-up biotechnology companies, and to academic laboratories illustrates this translational interface. Second, established infrastructure can strengthen competitiveness for federal funding by demonstrating operational readiness and scale. Third, coordinated statewide data collection may generate epidemiologic and health-economic insights relevant to policy decisions.

However, infrastructure alone does not guarantee translational success. Outcomes of interest, including validated non-invasive diagnostics, improved therapeutic strategies, and measurable changes in health equity, will determine the long-term value of this initiative.

## Scalability, alignment, and a path toward a federal network

Replication of the CT model in other states would require adaptation to local regulatory environments, healthcare systems, and demographic landscapes. Nonetheless, its core components—legislative mandate, harmonized phenotyping standards, public–academic partnership, formal oversight governance, and structured specimen dissemination—are conceptually transferable not only across U.S. states but also within international health systems seeking to strengthen women's health research infrastructure.

Following Connecticut's first of its kind mandated program, several states have taken legislative action to address endometriosis with varying scopes and success. In Virginia, HB 1918 (enacted unanimously in 2025) established a Women's Menstrual Health Program focused on provider and public education about endometriosis and related conditions (educational and awareness-focused, without an infrastructure component). In Massachusetts, in 2025, proposals to form a statewide endometriosis task force (currently under consideration) aim to improve clinical pathways and education but remain unfunded and without a formal biorepository strategy. In Washington State**,** legislative recommendations to improve endometriosis care and training have been advanced (bill proposals in 2024–2025) but are still in committee with mixed stakeholder support. These examples reflect growing recognition of endometriosis as a public health concern, but also illustrate divergent approaches and the absence of a consistent, infrastructure-oriented framework (Table 1).

**Table 1:** Legislative and policy landscape for endometriosis data infrastructure across the USA. Summary of enacted, proposed, and active legislative measures addressing endometriosis awareness, research infrastructure, and data sharing.

Jurisdiction	Policy (year)	Status	Primary focus	Infrastructure features	Notes
Connecticut	Public Act 22–33 (2022)	Enacted	Directed UConn Health, in consultation with JAX, to develop a plan for an endometriosis data and biorepository program	Planning mandate only (no operational repository); laid groundwork for statewide biorepository legislation	Precursor legislation; UConn Health/JAX planning report submitted to the Public Health Committee on Dec. 2022 was the foundation for PA 23–67
Connecticut	Public Act 23–67 (2023)	Enacted	Statewide endometriosis data and biorepository program	Mandated statewide biorepository; harmonized data collection; governance, oversight, and annual legislative reporting	Top performer: statutory, infrastructure-centred approach^a^
Connecticut	Public Act 26–13 (2026)	Enacted	Omnibus public health statute formally establishing the Endometriosis Working Group within the Commission on Women, Children, Seniors, Equity and Opportunity	Formalized working-group governance and legislative oversight; broadened mandate to study prevalence, diagnostic delay, workplace impact, and insurance coverage	Signed by Gov. Ned Lamont, May 14, 2026; extends CT’s statutory infrastructure beyond the original PA 23–67 mandate
Virginia	HB1918 Women’s Menstrual Health Program (2025)	Enacted	Provider and public education on menstrual disorders including endometriosis	Education and training programme	Policy success in awareness and training
Massachusetts	S.1564/H.2527/Amendment S.4 Endometriosis Task Force (2025–26 session)	Enacted	Proposed statewide task force on endometriosis	Task force recommendations only	Impact contingent on enactment
Washington	SB5985 "Concerning endometriosis" (2025–26)	Dead	Public education, resources for health care providers	Education and standards focus	Legislative momentum exists
Puerto Rico	Title 1 §5206	Enacted	Awareness campaigns and public education	None	Designates March as Endometriosis Month; directs outreach and awareness
Puerto Rico	PS174 Disability, Education for endometriosis patients	Proposed	Workplace and disability rights recognition for endometriosis patients, access to treatment and education	Legal recognition without infrastructure	Under discussion in legal and advocacy commentary
US Federal	H.R.6682 Endometriosis CARE Act	Proposed	National research funding, disparity studies, and awareness expansion	Would fund research; no federal biorepository mandate proposed	Illustrates emerging federal recognition of endometriosis as a research priority

a Connecticut’s Public Act 23–67 represents the only enacted statute in this review that mandates dedicated data and biospecimen infrastructure at the statewide level.

In contrast, CT's statute specifically created durable bipartisan-supported infrastructure with measurable deliverables and governance expectations as mandated by passed PA 26–13. This distinction matters: awareness campaigns and clinical task forces are critical, but they do not generate standardized biospecimens, harmonized data, and translational pipelines necessary for rigorous biomarker discovery, therapeutic development, and clinical trials. Rather than each state independently constructing isolated systems with variable definitions, protocols, and data standards, there is a strategic opportunity to integrate emerging state efforts into a coordinated, multi-jurisdiction network. Building on an already operational model such as EndoRISE, with harmonized sample and data platforms could accelerate collective impact, reduce duplication, and create a more compelling case for federal engagement.

Future evaluation frameworks should therefore include:

- Quantifiable scientific outputs linked to repository-derived specimens, including peer-reviewed publications and cross-institutional collaborations;
- Development, validation, and replication of diagnostic and prognostic biomarkers;
- Initiation of clinical trials informed by repository findings;
- Measurable reductions in diagnostic delay across integrated state networks;
- Demonstrable improvements in equitable access to diagnosis and specialty care.

Such multi-state metrics, coordinated through a shared governance and data infrastructure, would provide stronger evidence for the value of linking state-level biospecimen systems into a federally supported network. Without defined benchmarks and alignment mechanisms, assessment of policy-driven infrastructure will remain incomplete and fragmentation will persist.

## Conclusion

**Figure 1:**
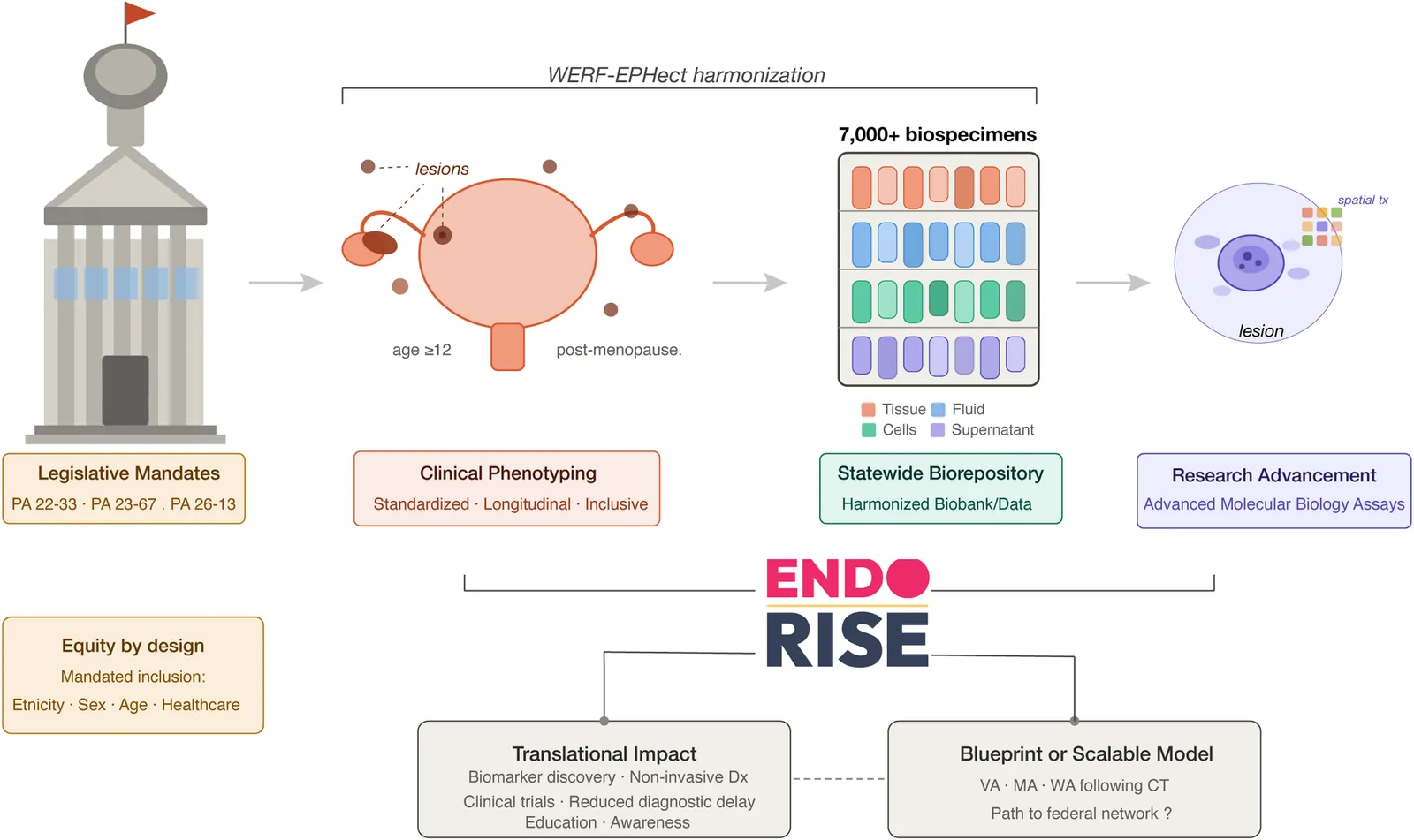
State-mandated infrastructure accelerates endometriosis research: the Connecticut EndoRISE model.

Endometriosis illustrates the broader challenge of advancing research in female-dominant conditions that have historically received limited structural investment. Connecticut's EndoRISE initiative represents a legislatively embedded attempt to align clinical care, harmonized biobanking, and translational research within a statewide framework. The program has progressed from planning to large-scale biospecimen collection and active sample dissemination to academic and biotechnology partners. Whether this infrastructure ultimately accelerates diagnostic innovation and improves outcomes will require continued evaluation. Nonetheless, the initiative provides an instructive case study of how state-level policy mechanisms may influence women's health research capacity (Figure 1).

## Data Availability

The original contributions presented in the study are included in the article/Supplementary Material, further inquiries can be directed to the corresponding author.
